# Supplementary material for: One Multilocus Genomic Variation Is Responsible for a Severe Charcot–Marie–Tooth Axonal Form
Source: Brain Sci. 2020 Dec 15;10(12):986. doi: 10.3390/brainsci10120986 (PMC7765239; doi:10.3390/brainsci10120986)
Supplement: Supplementary file 1 [file brainsci-10-00986-s001.pdf]

Supplementary Files

<u>Supplementary Table S1:</u> *92-gene panel used for NGS. It includes the 44 known CMT genes, 27 genes involved in HSN (Hereditary Sensitive Neuropathy) and HMN (Hereditary Motor Neuropathy) and 21 other genes of interest involved in neuropathies of differential diagnosis [R = recessively-inherited; D = dominantly-inherited].*

GENE	CMT1D	CMT2D	CMT1R	CMT2R	HMN D	HMNR	HSN D	HSN R	Other
AARS		X							
ABHD12									R
AIFM1				X					
ARHGEF10									D
ATL1							X		
ATL3							X		
ATP7A						X			
BICD2					X				
BSCL2					X				
CCT5								X	
CTDP1								X	
C12ORF65									R
DCAF8									D
DCTN1					X				
DHTKD1		X							
DNAJB2				X					
DNM2	X	X							
DNMT1							X		
DST								X	
DYNC1H1		X							
EGR2	X		X						
FAM134B								X	
FBLN5									D/R
FBXO38					X				
FGD4			X						
FIG4			X		X				
GAN									R
GARS		X			X				
GDAP1		X	X	X					
GJB1	X	X							
GJB3									D/R
GNB4	X	X							
HARS		X							
HINT1									R
HK1			X						
HSPB1		X			X				
HSPB3					X				
HSPB8		X			X				
IFRD1									D
IGHMBP2				X		X			
IKBKAP								X	
INF2	X	X							
KARS			X	X					
KIF1A								X	
KIF1B		X							
KIF5A									D
LITAF	X								
LMNA				X					
LRSAM1		X							
MARS		X							
MED25				X					
MFN2		X							
MPV17									R
MPZ	X	X	X						
MTMR2			X						
NDRG1			X						

NEFL	X	X							
NGF								X	
NTRK1								X	
PDK3		X							
PLEKHG5			X	X		X			
PMP22	X								
POLG									D/R
PRPS1				X					
PRX			X						
RAB7A		X							
REEP1					X				
SBF1			X						
SBF2			X						
SCN9A								X	
SCN10A							X		
SCN11A							X		
SEPT9									D
SETX					X				
SH3TC2			X						
SLC12A6									R
SLC5A7					X				
SMAD3									D
SOX10									D
SPTLC1							X		
SPTLC2							X		
SURF1			X						
TFG									D
TRIM2				X					
TRPV4		X			X				
TTR									D
TUBB3									D
UBQLN2					X				
VAPB									D
VCP									D
WNK1								X	
YARS	X	X							

<u>*Supplementary Table S2:*</u> *All genes included in the detected chromosome 16 duplication (from chr16:70185757 to chr16:70416579) (bp: base pair).*

Gene stable ID	Gene stable ID version	Gene name	Gene start (bp)	Gene end (bp)	Gene description
ENSG00000090857	ENSG00000090857.9	**PDPR**	70147529	70195203	pyruvate dehydrogenase phosphatase regulatory subunit
ENSG00000157335	ENSG00000157335.15	**CLEC18C**	70207225	70221264	C-type lectin domain family 18, member C
ENSG00000223496	ENSG00000223496.1	**EXOSC6**	70284134	70285833	exosome component 6
ENSG00000090861	ENSG00000090861.11	**AARS1**	70286198	70323446	alanyl-tRNA synthetase
ENSG00000157349	ENSG00000157349.11	**DDX19B**	70323566	70369186	DEAD (Asp-Glu-Ala-Asp) box polypeptide 19B
ENSG00000168872	ENSG00000168872.11	**DDX19A**	70380732	70407286	DEAD (Asp-Glu-Ala-Asp) box polypeptide 19A
ENSG00000157350	ENSG00000157350.8	**ST3GAL2**	70413338	70473140	ST3 beta-galactoside alpha-2,3-sialyltransferase 2
ENSG00000157353	ENSG00000157353.12	**FUK**	70488324	70514177	fucokinase
ENSG00000103051	ENSG00000103051.14	**COG4**	70514471	70557468	component of oligomeric golgi complex 4
ENSG00000189091	ENSG00000189091.8	**SF3B3**	70557691	70608820	splicing factor 3b, subunit 3, 130kDa
ENSG00000157368	ENSG00000157368.6	**IL34**	70613798	70694585	interleukin 34
ENSG00000132613	ENSG00000132613.10	**MTSS1L**	70695107	70719969	metastasis suppressor 1-like
